# Nutritional Quality and Price of Food Hampers Distributed by a Campus Food Bank: A Canadian Experience

**Published:** 2014-06

**Authors:** Mahsa Jessri, Arvin Abedi, Alexander Wong, Ghazaleh Eslamian

**Affiliations:** ^1^Department of Nutritional Sciences, Faculty of Medicine, University of Toronto, Toronto, ON, Canada; ^2^Co-Chair of the Statistics Committee, University of Alberta Campus Food Bank, Edmonton, AB, Canada; ^3^Research Institute for Endocrine Sciences (WHO Collaborating Center), Shahid Beheshti University of Medical Sciences, Tehran, Iran

**Keywords:** Food bank, Food insecurity, Nutrient adequacy, University, Canada

## Abstract

Food insecurity is a mounting concern among Canadian post-secondary students. This study was conducted to evaluate the content of food hampers distributed by University of Alberta Campus Food Bank (CFB) and to assess the cost savings to students, using these hampers. Contents of hampers distributed among 1,857 students and their dependants since 2006 were evaluated against Canada's Food Guide (CFG) recommendations and Dietary Reference Intakes (DRI). Hampers were aimed at serving university students and one to five members of their households located in Edmonton, Western Canada. One thousand eight hundred fifty-seven clients in Alberta, Canada, were included in the study. Although all hampers provided adequate energy, their fat and animal protein contents were low. Compared to the CFG recommendations, the requirements of milk and alternatives and meat and alternatives were not sufficiently met for clients using ≥3-person hampers. None of food hampers (i.e. one- to five-person hampers) met the DRI recommendations for vitamin A and zinc. Clients of CFB received Canadian dollar (CN$) 14.88 to 64.3 worth of non-perishable food items in one- to five-person hampers respectively. Hampers provided from the CFB need improvement. Nutrients missing from the food hampers could be provided from fresh fruits, vegetables, dairy, and meat products; however, these foods are more expensive than processed food items. The CFB provides a significant amount of savings to its clients even without considering the additional perishable donations that are provided to clients. Interpretation of our data required the assumption that all clients were consuming all of their hampers, which may not always be the case. Clients that do not fully consume their hampers may benefit less from the food bank.

## INTRODUCTION

According to the World Food Summit and the Canadian Government, food security requires that all people have physical and economic access to safe, sufficient and nutritious food at all times to meet their own nutritional needs and preferences and have an active and healthy life (1). Two main indicators of food security include availability of nutritionally-safe and adequate food and assured ability to acquire the food in socially-acceptable ways (i.e. without depending on emergency food supplies, stealing, scavenging, or other such strategies) (2). In contrast, food insecurity is described as limited or uncertain availability of nutritionally-safe and adequate food (2).

Food security is an important social determinant of health and is also related to income, which is a widely-recognized social determinant of health (3). Therefore, individuals under severe economic pressure, such as post-secondary students who are receiving financial aid, are a group at risk of food insecurity (4-8). Most commonly, the financial assistance that students receive is not sufficient for meeting their essential dietary requirements, which is a concern considering the rising number of university students (4-8). To address this serious obstacle, non-governmental food banks have been established, which are ad-hoc charitable organizations that distribute food items to families and individuals in need as a step towards fighting food insecurity and hunger (9).

In Canada, the first municipal food bank was established in 1981 in Edmonton, AB, through the realization of prevalent hunger in the community and, at the same time, high wastage of edible food (10). An ad-hoc committee was legally appointed to receive the first Canadian charter of food bank with the mission of providing the surplus from food industry to people experiencing food insecurity (10). Despite all the efforts, there are still people in Canada, whose lives are jeopardized by food insecurity, hunger, and food deprivation (11,12). Currently, over 650 food banks across Canada serve more than 820,600 clients, 95% of whom are food-insecure (13). According to the Canadian Community Health Survey (CCHS), a total of 2.3 million Canadians in 2004 suffered from food insecurity (14,15). In addition, approximately 3.7 million Canadians in 2005 were worried about having inadequate and/or poor-quality food (16). Food insecurity is associated with serious adverse health outcomes, such as anxiety, anaemia, chronic illnesses, depression, obesity, and overall poor health (17-19).

According to the latest statistics of the Canadian Association of Food Banks, currently 51 campus-based food banks exist nationwide (11). Campus Food Bank (CFB) at the University of Alberta, Edmonton, was opened in 1991 to address the growing problem of students’ hunger on campus and to distribute food items to all members of the university community, including students, staff, alumni, and their children. In particular, there has been a steady growth in the usage of CFB at the University of Alberta since its advent, with a two-fold increase in the number of hamper requests and people receiving food aids since 1993 (13). The CFB clients at the University of Alberta are supplied with hampers of non-perishable food items which are intended to last four days as well as various types and amounts of perishable food depending on the availability of these items.

However, previous studies have suggested that the food bank clients still experience hunger due to inadequacy of food hampers. In addition, food hampers provide inadequate amounts of some food-groups and nutrients mainly due to the restricted supply of perishable food (especially dairy products) that are dependent on unpredicted food donations (9,16,20,21). The quality of food hampers has also been questioned in previous studies, with the vast majority of hampers reported to have at least one unsafe item (damaged or past due date) (9). Generally, campus food banks are less studied, and the only study evaluating hamper menus at the University of Alberta in 2003 reported the problem of nutrient inadequacy among CFB hampers (4,5). The CFB hamper menus were revised in 2010 and 2011 to supply a more economic and nutritious meal plan. The aims of the present research were, therefore, to: (a) evaluate and compare the nutritional adequacy of food hampers distributed in 2006, 2010, and 2011 among CFB clients in relation to Canada's Food Guide (CFG) and dietary reference intakes (DRI) and (b) estimate the cost-savings to students, using CFB at the University of Alberta.

## MATERIALS AND METHODS

CFB provides each client with one of the five sizes of food hampers worth of four days of food. The amounts and types of perishable items in all hampers are similar, except for eggs which are provided 4 per person and milk which is 1 litre for one- and two-person hampers and 2 litre for ≥3-person hampers. However, since provision of perishable items depends mainly on donations, the content is largely variable. Overall, 619 clients received one-person hampers while 426, 261, 356, and 195 clients received two-, three-, four- and five-person hampers respectively (n=1,857). For hampers that are intended to be shared with children (2-17 years), peanut butter and yogurt are also considered.

Hampers have historically been designed by the discretion of the Executive Director of the Campus Food Bank. The decision is made based on the availability of items as well as their cost. The hamper menu is subject to change anytime during the year to allow flexibility. Changes made to the menu must maintain the four-day volume requirement. Each hamper has been designed with the expressed intent to provide enough food for four days. The way the Campus Food Bank has determined four days worth of food, is not through any specific measurement method, i.e. caloric content, weight, cost, etc. It is simply what past executive directors felt met approximately four days worth of food, assuming an average person is consuming the food with an average appetite. The amount is then scaled linearly as more individuals are consuming a single hamper request.

Content of each hamper was recorded and Food Processor SQL (version 9.1.0, ESHA Research, Salem, OR) was used in analyzing the dietary content, energy, and nutrients of one- to five-person food hampers, using Canada Nutrient File. We also specified the number of servings received from each food-group according to the CFG (22). In total, 5 sizes of hampers were analyzed for 3 years (2006, 2010, and 2011) and, for each hamper, comparisons were made between nutrient contents of hampers with and without perishable food items. Since one- to five-person food hampers provided food items for 4 days, nutrient values were divided by four to reflect daily intake values (Table 1-4). Since CFB does not distinguish between hampers provided to males, females, or children, the strictest guidelines were employed to consider an adult male as a reference for all nutrient intake analyses (6,23).

In September 2011, non-perishable hamper items were priced at three different supermarkets closest to the University of Alberta campus, using household brands and non-sale prices. The staff and managers of the CFB were consulted throughout the data analyses and interpretation in order to ensure accuracy of findings. Permissions were granted from the CFB directors and authorities at the University of Alberta, Canada, for this research.

## RESULTS

Generally, data for 1,025 unique CFB clients were analyzed (60% females, 40% males). Mean±SD age of clients in 2011 was 27.98±8.07 years, 18.4% of clients were married, and 91.7% were full-time students (data not shown). Undergraduate and graduate students constituted 67.9% and 22.7% of the total clients while post-doctoral fellows and open studies/after degree/staff/alumni clients constituted the remaining 0.1% and 6.9% respectively. Between 2003 and 2010, the hamper requests have increased from 8.3% to 17.9% respectively (data not shown).

**Table 1.** Contents of a one-person food bank hamper with four days worth of food in 2006, 2010, and 2011*

Non-perishable staple	2006	2010	2011
	(Quantity)	(Quantity)	(Quantity)
Cans of beans	2	2	2
Cans of tuna, salmon, turkey, or other meat	2	2	2
Cans of soup (or dried soup)	2	824 mL	824 mL
Cans of vegetables (e.g. mixed, corn, etc.)	2	2	2
Cans of fruit	509 g	509 g	509 g
Pasta or tomato sauce	398 mL	398 mL	398 mL
Macaroni and cheese dinner mix	1	1	1
Pasta	500 g	500 g	500 g
Rice	500 g	500 g	500 g
Rolled oats	500 g	500 g	500 g
Juice	1 L	1 L	1 L
Powdered milk	300 g	–	–
Box/bag of cereal	–	–	500 g
Perishable item	(Quantity)	(Quantity)	(Quantity)
Eggs	4	4	0
Medium apple	1	1	0
Medium orange	1	1	0
Raw carrot	1	1	0
Raw yellow onion	1	1	0
Bagels, whole wheat	6	6	0
Tofu dessert/per household	0	0	4
Caesar salad kits/per household	0	0	4
Jello Cheesecake dessert/per household	0	0	1
Cheese brick/per household	0	0	1
Frozen Shanghai Noodles/per household	0	0	2
Frozen bag of Hash browns/per household	0	0	1
Large pack of frozen ham/per household	0	0	1
Containers of broccoli and celery/per household	0	0	2
Mini watermelon/per household	0	0	1
Bag of carrots/per household	0	0	1
Bag of Zucchini/per household	0	0	1
Bag of peppers/per household	0	0	1
Milk	0	0	1 L

*All commercial names are excluded but were used for coding and nutrient analysis

**Table 2.** Contents of a two-person food bank hamper with four days worth of food in 2006, 2010, and 2011*

Non-perishable staple	2006	2010	2011
	(Quantity)	(Quantity)	(Quantity)
Cans of beans	3	3	3
Cans of tuna, salmon, turkey, or other meat	3	3	3
Cans of soup (or dried soup)	3	1,108 mL	1,108 mL
Cans of vegetables (e.g. mixed, corn, etc.)	3	3	3
Cans of fruit	764 g	764 g	764 g
Pasta or tomato sauce	398 mL	398 mL	398 mL
Macaroni and cheese dinner mix	2	2	2
Pasta	500 g	500 g	500 g
Rice	750 g	750 g	750 g
Rolled oats	500 g	500 g	500 g
Juice	1 L	1 L	1 L
Powdered milk	500 g	–	–
Box/bag of cereal	–	–	500 g
Peanut butter	500 g	500 g	500 g
Perishable item	(Quantity)	(Quantity)	(Quantity)
Yogurt	250 g	0	0
Eggs	8	8	8
Medium apple	1	1	0
Medium orange	1	1	0
Raw carrot	1	1	0
Raw yellow onion	1	1	0
Bagels, whole wheat	6	6	0
Tofu dessert/per household	0	0	4
Caesar salad kits/per household	0	0	4
Jello Cheesecake dessert/per household	0	0	1
Cheese brick/per household	0	0	1
Frozen Shanghai Noodles/per household	0	0	2
Frozen bag of Hash browns/per household	0	0	1
Large pack of frozen ham/per household	0	0	1
Containers of broccoli and celery/per household	0	0	2
Mini watermelon/per household	0	0	1
Bag of carrots/per household	0	0	1
Bag of Zucchini/per household	0	0	1
Bag of peppers/per household	0	0	1
Milk	0	0	1 L

*All commercial names are excluded but were used for coding and nutrient analysis

Table 1-5 present the contents of one- to five-person hampers provided for four days in 2006, 2010, and 2011. Generally, with regard to non-perishable items, all hampers in 2006 included 300-1,000 g of powdered milk which was excluded from the 2010 and 2011 hampers. Another minor difference was that, in 2011, a 500-g box of cereal was included in 2-person hampers and 250 g and 500 g additional rice was added to four- and five-person hampers respectively. The most dramatic change during 2006 to 2011 has been the improvement in amounts and types of perishable items included in the 2011 hampers (Table 1-5). Overall, as Table 6-10 indicate, the nutritional quality of all hamper-sizes improved significantly in 2006, 2010, and 2011 if perishable items were included. For instance, in 2011, the one-person hamper provided a range of 2,668-3,251 kcal/day depending on whether perishable items were included, with approximately 70.9% of energy derived from carbohydrates, 19.3% from protein, and 9.7% from fat. The energy contents increased monotonously between two- and three-person hampers, although calorie contents of four- and five-person hampers decreased. For one-, two- and three-person hampers, the requirements of all food-groups were met, and inclusion of non-perishable items resulted in excess provision of grain products. For two-, three- and five-person hampers, meat and alternatives were provided more than the recommended daily amount. In addition, the milk and alternatives requirements were not met in four- and five-person hampers.

**Table 3.** Contents of a three-person food bank hamper with four days worth of food in 2006, 2010, and 2011^*^

Non-perishable staple	2006	2010	2011
	(Quantity)	(Quantity)	(Quantity)
Cans of beans	4	4	4
Cans of tuna, salmon, turkey, or other meat	4	4	4
Cans of soup (or dried soup)	4	1,648 mL	1,648 mL
Cans of vegetables (e.g. mixed, corn, etc.)	4	4	4
Cans of fruit	1.02 kg	1.02 kg	1.02 kg
Pasta or tomato sauce	796 mL	796 mL	796 mL
Macaroni and cheese dinner mix	3	3	3
Pasta	800 g	800 g	800 g
Rice	1 kg	1 kg	1 kg
Rolled oats	500 g	500 g	500 g
Juice	1 L	1 L	1 L
Powdered milk	500 g	–	–
Box/bag of cereal	500 g	500 g	500 g
Peanut butter	500 g	500 g	500 g
Perishable item	(Quantity)	(Quantity)	(Quantity)
Eggs	12	12	12
Medium apple	3	3	0
Medium orange	3	3	0
Raw carrot	3	3	0
Raw yellow onion	3	3	0
Bagels, whole wheat	6	6	0
Tofu dessert/per household	0	0	4
Caesar salad kits/per household	0	0	4
Jello Cheesecake dessert/per household	0	0	1
Cheese brick/per household	0	0	1
Frozen Shanghai Noodles/per household	0	0	2
Frozen bag of Hash browns/per household	0	0	1
Large pack of frozen ham/per household	0	0	1
Containers of broccoli and celery/per household	0	0	2
Mini watermelon/per household	0	0	1
Bag of carrots/per household	0	0	1
Bag of Zucchini/per household	0	0	1
Bag of peppers/per household	0	0	1
Milk	0	0	2 L

*All commercial names are excluded but were used for coding and nutrient analysis

With regard to micronutrients, vitamin A and zinc contents in all hampers were inadequate, and most hampers provided borderline vitamin C and folate for CFB clients. The one-person hamper contained CN$14.88 worth of non-perishable items while the two-, three-, four- and five-person hampers contained food valued at CN$ 21.77, 36.64, 51.32, and 64.3 respectively.

## DISCUSSION

Findings from this research highlight several concerning aspects of nutritional quality of hampers distributed by CFB at the University of Alberta. Although the energy content of hampers was seemingly adequate, the relatively high servings of carbohydrates in the form of grain products decreased the percentage of calories provided by fat. However, the fat content in hampers prepared for children was higher due to the inclusion of peanut butter. In addition, vitamin A and zinc contents of all sizes of hampers were inadequate.

**Table 4.** Contents of a four-person food bank hamper with four days worth of food in 2006, 2010, and 2011^*^

Non-perishable staple	2006	2010	2011
	(Quantity)	(Quantity)	(Quantity)
Cans of beans	5	5	5
Cans of tuna, salmon, turkey, or other meat	5	5	5
Cans of soup (or dried soup)	5	1,932 mL	1,932 mL
Cans of vegetables (e.g. mixed, corn, etc.)	5	5	5
Cans of fruit	1.27 kg	1.27 kg	1.27 kg
Pasta or tomato sauce	1,078 mL	1,078 mL	1,078 mL
Macaroni and cheese dinner mix	4	4	4
Pasta	1 kg	1 kg	1 kg
Rice	1 kg	1 kg	1.25 kg
Rolled oats	1 kg	1 kg	1 kg
Juice	2 L	2 L	2 L
Powdered milk	800 g	–	–
Box/bag of cereal	–	500 g	500 g
Peanut butter	500 g	500 g	500 g
Perishable item	(Quantity)	(Quantity)	(Quantity)
Eggs	16	16	16
Medium apple	3	3	0
Medium orange	3	3	0
Raw carrot	3	3	0
Raw yellow onion	3	3	0
Bagels, whole wheat	6	6	0
Tofu dessert/per household	0	0	4
Caesar salad kits/per household	0	0	4
Jello Cheesecake dessert/per household	0	0	1
Cheese brick/per household	0	0	1
Frozen Shanghai Noodles/per household	0	0	2
Frozen bag of Hash browns/per household	0	0	1
Large pack of frozen ham/per household	0	0	1
Containers of broccoli and celery/per household	0	0	2
Mini watermelon/per household	0	0	1
Bag of carrots/per household	0	0	1
Bag of Zucchini/per household	0	0	1
Bag of peppers/per household	0	0	1
Milk	0	0	2 L

*All commercial names are excluded but were used for coding and nutrient analysis

Of concern was also the fact that major portion of iron was obtained from beans and peanut butter which are non-haem and may jeopardize iron sufficiency in those dependent on CFB for long periods of time. In addition, high fibre content in hampers provided by the CFB increased the risk of iron deficiency since fibre and non-nutrient components of plants (e.g. phytate, lectins, tannins, saponins) interact with nutrients (such as iron and zinc) and may reduce their absorption. Specifically, phytic acid (phytate) is a six-carbon compound that is found in the seed-coat of grains and legumes and can bind metal ions, especially calcium, copper, iron, and zinc and reduce their bioavailability (24). Lipids and fats should constitute about 34% of the energy in human diet and, therefore, inadequate fat content in hampers may have several detrimental effects. Dietary fat is important for absorption, digestion, and transportation of fat-soluble vitamins and phytochemicals, such as lycophenes and carotenoids (24). Dietary fat facilitates digestion through depressing gastric secretions, slowing gastric emptying and stimulating biliary and pancreatic flow.

**Table 5.** Contents of a five-person food bank hamper with four days worth of food in 2006, 2010, and 2011^*^

Non-perishable staple	2006	2010	2011
	(Quantity)	(Quantity)	(Quantity)
Cans of beans	6	6	6
Cans of tuna, salmon, turkey or other meat	6	6	6
Cans of soup (or dried soup)	6	2,472 mL	2,472 mL
Cans of vegetables (e.g. mixed, corn, etc.)	6	6	6
Cans of fruit	1.53 kg	1.53 kg	1.53 kg
Pasta or tomato sauce	1,078 mL	1,078 mL	1,078 mL
Macaroni and cheese dinner mix	5	5	5
Pasta	1.5 kg	1.5 kg	1.5 kg
Rice	1 kg	1 kg	1.5 kg
Rolled oats	–	1 kg	1 kg
Juice	2 L	2 L	2 L
Powdered milk	1 kg	–	–
Box/bag of cereal	1 kg	1 kg	1 kg
Peanut butter	1 kg	1 kg	1 kg
Perishable item	(Quantity)	(Quantity)	(Quantity)
Eggs	20	20	20
Medium apple	5	5	0
Medium orange	5	5	0
Raw carrot	5	5	0
Raw yellow onion	5	5	0
Bagels, whole wheat	6	6	0
Tofu dessert/per household	0	0	4
Caesar salad kits/per household	0	0	4
Jello Cheesecake dessert/per household	0	0	1
Cheese brick/per household	0	0	1
Frozen Shanghai Noodles/per household	0	0	2
Frozen bag of Hash browns/per household	0	0	1
Large pack of frozen ham/per household	0	0	1
Containers of broccoli and celery/per household	0	0	2
Mini watermelon/per household	0	0	1
Bag of carrots/per household	0	0	1
Bag of Zucchini/per household	0	0	1
Bag of peppers/per household	0	0	1
Milk	0	0	2 L

*All commercial names are excluded but were used for coding and nutrient analysis

Another issue observed in this study was inadequacy of animal proteins in hampers, even though the total protein content was within the recommended range. Protein quality depends on the bioavailability of all necessary amino acids and animal proteins score higher than vegetable proteins (from legumes and beans) (24). Since vegetable protein is encased in carbohydrate, it is less available to digestive enzymes compared to animal proteins. Particularly, some plants (e.g. soy) contain enzymes that require heat activation before consumption in order to be digested. Generally, diets that are based on plant foods do not have enough of limiting amino acids to provide substrate for protein synthesis. However, one suggestion for improving protein quality is to add another plant protein that contains an excess of the limiting amino acid to complement the protein combination and facilitate protein synthesis. For instance, combining grains with legumes, grains with dairy, and legumes with seeds would provide all essential amino acids (24).

Low vitamin A and zinc contents of hampers are also of note, especially for long-term consumers of CFB hampers. Low intakes of vitamin A can result in several complications, including impaired vision (e.g. night blindness, nyctalopia), anaemia, abortion, impaired immune system, poor growth, and bone defects. Zinc deficiency also has clinical signs, such as short stature, anaemia, hypogonadism, immunologic defects, alopecia, and skin lesions.

**Table 6.** Nutrient content and servings from each food-group of a one-person food hamper for an adult in 2006, 2010, and 2011^1^

Nutrient	Dietary reference intake	2006		2010		2011	
		Staple	Staple and perishable item	Staple	Staple and perishable item	Staple	Staple and perishable item
Energy (kcal)		2,458	3,061	2,468	3,071	2,668	3,251
% energy protein	10-35	19.0	19.1	18.9	19.0	19.3	19.3
% energy carbohydrate	45-65	72.0	72.0	73.0	73.0	71.0	70.9
% energy fat	20-35	9.0	8.9	9.1	9.0	9.7	9.8
Folate (μg)	400	538.0	578.0	540.0	581.2	597.3	625.5
Vitamin C (mg)	75*-90	112	138	110	141	119	149
Vitamin A (RE)	700*-900	426.5	729.0	425.8	789.2	436.3	801.5
Calcium (mg)	1,000	1,438	1,541	1,441	1,561	1,591	1,678
Iron (mg)	8-18*	25.0	32.3	25.1	33.5	26.0	39.5
Zinc (mg)	8*-11	6.2	6.6	6.3	6.8	6.9	8.5
Dietary fibre (g)	25*-38	41	53	42.1	57	44.1	61.3
Food-group	Recommended number of foodguide servings						
Grain products	5-12	11.4	14.4	11.7	14.8	12.1	14.5
Vegetables and fruits	5-10	6.1	7.3	6.2	7.3	6.2	7.6
Milk products	2-4	2.5	2.5	2.5	2.5	2.7	3.1
Meat and alternatives	2-3	2.2	3.3	2.2	3.2	2.7	3.8

^1^Mean of four-day supply based on whether hamper contained staple items only or staples plus perishables;

*Females

**Table 7.** Nutrient content and servings from each food-group of a two-person food hamper for an adult in 2006, 2010, and 2011^1^

Nutrient	Dietary reference intake	2006		2010		2011	
		Staple	Staple and perishable item	Staple	Staple and perishable item	Staple	Staple and perishable item
Energy (kcal)		2,493	3,094	2,501.5	3,103	2,617	3,262
% energy protein	10-35	18.2	17.7	18.3	17.8	18.4	18.2
% energy carbohydrate	45-65	63.0	65.1	63.1	65.0	62.1	64.1
% energy fat	20-35	18.8	17.2	18.6	17.2	19.5	17.7
Folate (μg)	400	413.5	405.1	422.6	453.3	442.1	451
Vitamin C (mg)	75*-90	72.75	103.5	74.2	105.2	86.5	111
Vitamin A (RE)	700*-900	287.5	592.1	293.6	593.5	302.2	676.2
Calcium (mg)	1,000	1,377	1,461	1,381	1,463.5	1,424.2	1,476
Iron (mg)	8-18*	23	28.8	23.7	30.1	29.7	35.8
Zinc (mg)	8*-11	4.4	8.3	6.9	8.4	9.25	10.15
Dietary fibre (g)	25*-38	39.75	49.5	38.25	51	47.5	64.5
Food-group	Recommended number of foodguide servings						
Grain products	5-12	10.8	12.5	11.6	12.9	12.8	13.1
Vegetables and fruits	5-10	6.4	7.0	6.5	7.0	6.7	7.9
Milk products	2-4	2.1	2	2.1	2.3	2.4	2.8
Meat and alternatives	2-3	4.6	5.1	5.3	5.0	5.0	5.4

^1^Mean of four-day supply based on whether hamper contained staple items only or staples plus perishables;

*Females

**Table 8.** Nutrient content and servings from each food-group of a three-person food hamper for an adult in 2006, 2010, and 2011^1^

Nutrient	Dietary reference intake	2006		2010		2011	
		Staple	Staple and perishable item	Staple	Staple and perishable item	Staple	Staple and perishable item
Energy (kcal)		2,738	3,358	2,744	3,404	3,012	3,309
% energy protein	10-35	18.1	17.8	18.2	17.8	18.3	18.2
% energy carbohydrate	45-65	63.1	65.0	63.2	65.0	62.2	64.1
% energy fat	20-35	18.8	17.2	18.6	17.2	19.5	17.7
Folate (μg)	400	428.5	421.1	439.6	472.3	462.05	478
Vitamin C (mg)	75*-90	83.7	115.5	87.2	122.1	105.5	136
Vitamin A (RE)	700*-900	294.5	620.1	316.6	616.5	334.2	734.2
Calcium (mg)	1,000	1,402	1,496	1,412	1,495.5	1,457.2	1,531
Iron (mg)	8*-18*	21	25.8	22.1	29.8	33.5	39.7
Zinc (mg)	8*-11	4.5	8.7	7.1	8.3	11.3	14.3
Dietary fibre (g)	25*-38	38.5	47.8	37.5	48	53.2	66.5
Food-group	Recommended number of foodguide servings						
Grain products	5-12	11.5	13.1	12.4	13.0	12.6	13.1
Vegetables and fruits	5-10	6.3	7.1	6.3	7.2	6.7	7.9
Milk products	2-4	1.9	2.0	2.0	2.1	2.4	2.6
Meat and alternatives	2-3	4.7	4.8	4.9	5.0	5.8	6.4

^1^Mean of four-day supply based on whether hamper contained staple items only or staples plus perishables;

*Females

**Table 9.** Nutrient content and servings from each food-group of a four-person food hamper for an adult in 2006, 2010, and 2011^1^

Nutrient	Dietary reference intake	2006		2010		2011	
		Staple	Staple and perishable item	Staple	Staple and perishable item	Staple	Staple and perishable item
Energy (kcal)		2,611	3,221	2,636	3,229	2,887	3,101
% energy protein	10-35	18.2	18.1	17.7	17.8	18.2	18.3
% energy carbohydrate	45-65	63.0	63.1	65.1	65.0	64.1	64.2
% energy fat	20-35	18.8	18.8	17.2	17.2	17.7	17.5
Folate (μg)	400	398.5	401.5	414.5	448.5	431.3	451.3
Vitamin C (mg)	75*-90	78.5	103.3	81.6	118.5	93.5	124.5
Vitamin A (RE)	700*-900	277.3	601.8	302.5	598.5	305.8	711.5
Calcium (mg)	1,000	1,294	1,305	1,298	1,335	1,432	1,485
Iron (mg)	8-18*	19.5	23.8	20.1	27.8	31.5	37.7
Zinc (mg)	8*-11	3.8	4.9	5.8	6.9	10.1	13.1
Dietary fibre (g)	25*-38	35.1	44.3	34.3	44.5	49.1	67.3
Food-group	Recommended number of foodguide servings						
Grain products	5-12	11	12.0	11.3	12.2	12.2	12.5
Vegetables and fruits	5-10	6.1	6.8	6.2	6.9	6.4	7.7
Milk products	2-4	1.3	1.8	1.7	1.9	2.1	2.5
Meat and alternatives	2-3	4.3	4.5	4.3	4.8	5.7	6.3

^1^Mean of four-day supply based on whether hamper contained staple items only or staples plus perishables;

*Females

**Table 10.** Nutrient content and servings from each food-group of a five-person food hamper for an adult in 2006, 2010, and 2011^1^

Nutrient	Dietary reference intake	2006		2010		2011	
		Staple	Staple and perishable item	Staple	Staple and perishable item	Staple	Staple and perishable item
Energy (kcal)		2,501	3,111	2,596	3,189	2,787	3,041
% energy protein	10-35	18.2	18.1	17.7	17.8	18.3	18.0
% energy carbohydrate	45-65	63.0	63.1	65.1	65.0	62.2	64.9
% energy fat	20-35	18.8	18.8	17.2	17.2	19.5	17.1
Folate (μg)	400	378.4	389.3	400.3	429.7	419.3	429.3
Vitamin C (mg)	75*-90	64.5	91.3	69.6	98.5	78.5	106.5
Vitamin A (RE)	700*-900	261.2	591.9	291.2	582.5	295.8	697.4
Calcium (mg)	1,000	1,184	1,201	1,158	1,215	1,342	1,375
Iron (mg)	8-18*	15.5	19.7	16.9	25.1	28.5	32.7
Zinc (mg)	8*-11	2.9	3.7	4.4	5.8	9.4	11.8
Dietary fibre (g)	25*-38	30.9	39.1	32.7	40.5	41.5	62.3
Food-group	Recommended number of foodguide servings						
Grain products	5-12	10.8	11.4	11.3	11.5	11.7	12.2
Vegetables and fruits	5-10	5.7	6.2	5.9	6.1	6.4	7.7
Milk products	2-4	1.1	1.4	1.6	1.8	1.7	2.0
Meat and alternatives	2-3	3.9	4.1	4.3	4.8	5.3	6.1

^1^Mean of four-day supply based on whether hamper contained staple items only or staples plus perishables;

*Females

This is even more important in this study since high fibre consumption may prevent zinc absorption through chelating with this mineral in the intestine (24).

Although perishable items improved the quality of hampers remarkably, these were only provided when donations were available. Not counting the perishable items, a one-person hamper contained CN$14.88 worth of food, which is a substantial cost-saving to students in the long term (5). According to a previous study conducted on CFB hamper prices, a one-person hamper with perishable items yielded CN$ 30.63 savings to a university student while the two-person hamper contained CN$ 58.02 worth of food.

### Limitations

This study has several limitations which may have implications for interpretation of results. First, we assumed that all items in a food hamper would be consumed within four days while, in practice, there might be some food items that are not favoured and, therefore, not entirely consumed or totally rejected. In addition, we made the arbitrary assumption that the hampers that were shared with a child would be consumed equally by children and adults since CFB does not make any distinctions between clients by age and gender categories. However, apportioning might not be in accordance with individuals’ needs as previous studies have suggested that unprivileged parents might sacrifice their own needs in order to feed their children (25,26).

### Conclusions

Overall, for clients who rely on CFB for food items, food hampers do not provide a healthful diet, especially with regard to micronutrients contained in milk and alternatives as well as the fat-soluble vitamins. More attention should be directed towards the dietary intakes of university students and their children living with food insecurity.

Collaboration of dietitians with food bank staff in planning and designing comprehensive hamper menus and educating clients on healthful eating on a limited budget might ensure improved nutritional quality of university students confronting food insecurity.

Dietitians could train food bank staff on principles of a healthful diet and provide them with nutritional policy tips in order to enable them to meet their clients’ needs more efficiently. It is suggested that food bank staff secure partnerships with stakeholders and donors to meet the requirements of the new hamper menu. Furthermore, to increase the volume of food sources, the staff should work to secure open communication and transportation with other food services as a previous report has shown that some foods may be put to waste by a food assistance programme while other ones within the same city may be in desperate need of the same foods. Governmental campaigns promoting donations of perishable food items may also be an efficient solution to meeting nutritional needs of food bank clients.
